# Switch-over Study with Fast-acting Insulin Aspart Showing Lower Glycemic Variability in Type 2 Diabetics with Stage 4 Chronic Kidney Disease: A Case Series

**DOI:** 10.7759/cureus.6344

**Published:** 2019-12-11

**Authors:** Sayak Roy, Mridul Bera, Guruprasad Bhattacharya, Maneesha Khalse

**Affiliations:** 1 Internal Medicine, Calcutta Medical Research Institute Hospital, Kolkata, IND; 2 Internal Medicine, Narayana Super Speciality Hospital, Howrah, IND; 3 Medical Services, Medical Affairs Division Lupin Limited, Mumbai, IND

**Keywords:** fast-acting insulin aspart, chronic kidney disease, glycemic variability

## Abstract

Background

Despite conventional insulin treatment being considered an effective approach for glycemic regulation during renal dysfunction, there is still a major clinical need for better insulin therapy to stabilize fluctuations in glucose levels.

Aim

The aim of this study was to assess the impact of mealtime fast-acting insulin aspart therapy on glycemic variability as compared to regular human insulin therapy in advanced chronic kidney disease (CKD) patients with type 2 diabetes (T2D).

Methods

Data from eight patients were retrospectively collected after analyzing 57 patients' data between July 2019 and October 2019. All T2D patients with stage 4 CKD were switched to mealtime fast-acting insulin aspart on account of recurrent hypoglycemic events. The continuous glucose monitoring data of the first four days were analyzed to calculate the mean amplitude of glucose excursions (MAGE) as well as five other glycemic variability indices, namely, standard deviation, mean, continuous overall net glycemic action, average daily risk range, and J index.

Results

The primary endpoint of 24-h MAGE significantly decreased from 7.01 ± 2.59 to 4.19 ± 1.06 mg/dL (p = 0.012) when short-acting regular human insulin (RHI) therapy was replaced with mealtime fast-acting insulin aspart therapy. However, no significant change was observed in 24-h mean glucose levels and other indices of glucose variability. Significant reduction in 24-h and night-time hypoglycemic events was reported in patients after therapy switch during the four-day follow-up period.

Conclusions

The present study demonstrated an improvement in glycemic variability with the administration of fast-acting insulin aspart as compared to RHI, suggesting that modern bolus insulin replacement might prove to be a useful therapeutic strategy in type 2 diabetes patients with advanced CKD. Further clinical studies will be required to confirm the benefits of this therapeutic approach.

Relevance for patients

The safety and effectiveness of fast-acting insulin aspart in CKD patients have not yet been established. The clinical effectiveness and better safety profile of newer mealtime insulin therapy may prompt a reconsideration of its use in patients with an advanced stage of renal dysfunction, leading to better adherence and improved quality of life.

## Introduction

Chronic hyperglycemia is the primary risk factor for the development of complications in diabetes mellitus (DM) patients; however, frequent or large glucose fluctuations may independently contribute to diabetes-related complications. Both postprandial glycemic excursions and the frequency of hypoglycemic events are shown to be associated with elevated cardiovascular events [1]. The diminished frequency of or absent glycemic autoregulation or shortfalls of insulin availability is hypothesized to be the major etiological factor for glycemic variability (GV). The correction of GV could act as a potential target approach to safely reduce the mean blood glucose level and determine its direct effects on vascular complications in diabetes patients. Modern diabetes management modalities, including glucagons like peptide-1 receptor agonist-based therapy, novel types of insulin, modern insulin pumps, and bariatric surgery, are associated with a significant reduction of GV. The mean amplitude of glucose excursion (MAGE) was designed to evaluate mealtime-related glucose excursions. Intermittent high blood glucose exposure has been shown to have a deleterious effect in previous experimental studies [2]. Increasing evidence suggests that long-term glucose variability might promote the renal complications of diabetes independent of hyperglycemia [3]. Regardless of the fact that insulin is considered the best therapeutic agent, especially in patients with severe renal dysfunction and end-stage renal disease, managing hyperglycemia with intensive glucose regulation is challenging due to altered insulin clearance and renal glucose handling, changed pharmacokinetic parameters, and dosing adjustment in these patients. However, modern insulin therapies, like fast-acting insulin aspart, designed to replicate normal physiology, aim to both lower A1C levels and stabilize glucose fluctuations. Fast-acting insulin aspart therapy is a modified version of insulin aspart therapy containing niacinamide and l-arginine, which leads to faster initial insulin absorption. This consequently leads to an earlier onset of action and greater early glucose-lowering effect as compared to insulin aspart. Injectable forms of fast-acting insulin aspart were first approved in the United States in 2017. Information about the choice of various preparations of insulin and about differences in insulin profiles along with their impact on glycemic variability is still limited because only a few studies have examined patients with advanced renal insufficiency. The present study was conducted with the aim to assess the impact of mealtime fast-acting insulin aspart therapy on GV after switching from regular human insulin in advanced chronic kidney disease patients with type 2 diabetes.

## Materials and methods

Eight patients were retrospectively analyzed from the outpatient clinics from July 2019 to October 2019. All T2D diagnosed patients, with stage 4 CKD (CKD-Epidemiology Collaboration (EPI)) and were receiving stable basal-bolus insulin therapy for more than three months were switched to mealtime faster insulin aspart on unit-to-unit basis due to capillary blood glucose (CBG) documented recurrent hypoglycemic events (60 mg/dL or less) as the bolus part of intensive therapy during the follow-up period. The target fasting glucose level (130 mg/dL) was maintained by basal insulin glargine (16 ± 4.56 units) with no oral hypoglycemic agents in the background and no recent history of hospitalization within the last three months. Table 1 shows the patient characteristics of the studied group.

**Table 1:** Patient characteristics SD: standard deviation, N: number of values counted, HbA1c: glycated hemoglobin A1c, eGFR: estimated glomerular filtration rate, RHI: regular human insulin, DM: diabetes mellitus, CKD EPI: Chronic Kidney Disease Epidemiology Collaboration, *Mean ± SD

Patient profiles (n = 8)	
Gender ratio (Male:Female)	1:1
Age (years)	65.63 ± 4.61*
Height (cm)	161.5 ± 4.07*
Body weight (kg)	68.0 (± 3.97) *
Body mass index (kg/m^2^)	25.4 (± 1.25)*
Duration of DM (years)	11.13 ± 0.83*
HbA1c (%)	8.2 (0.44)*
eGFR (CKD EPI) mL/min/1.73 m^2^	22 ± 4.21*
Total daily RHI (IU/kg)	24.25 ± 7.14* (0.35 U/kg)
Total daily insulin glargine dose (IU/kg)	16 ± 4.56 at 9 pm (0.24 U/kg)
Total daily Fiasp dose (IU/kg)	20.8 ± 5.0* (0.29 U/kg)

Continuous glucose monitoring (CGM) (iPro2; Medtronic PLC, Dublin, Ireland) was conducted after 10 days of stable glucose maintenance as per the five-point CBG record (fasting, pre-lunch, two-hour post-lunch, pre-dinner, and two-hour post-dinner). A CGM sensor was inserted into the subcutaneous abdominal fat tissue and calibrated according to the standard Medtronic iPro2 operating guidelines. While wearing the CGM, the patients checked their blood glucose levels with a self-monitoring blood glucose device at least four times per day.

The CGM data of the first four days of each patient were analyzed with the EasyGV Version 9.0.R2 software (Nuffield Department of Primary Care Health Sciences, Oxford, UK) to calculate MAGE as well as five other glycemic variability indices, namely, standard deviation (SD), mean, continuous overall net glycemic action, average daily risk range, and J index.

Since the data was of a retrospective nature and all the patients had provided written informed consent, no ethical committee approval was needed. All the protocols were designed as per the guidelines of the Declaration of Helsinki.

## Results

The primary endpoint of 24-h MAGE significantly decreased from 7.01 ± 2.59 to 4.19 ± 1.06 mg/dL (p = 0.012) when rapid-acting regular human insulin (RHI) was replaced by mealtime fast-acting insulin aspart. However, no significant change was observed in 24-h mean glucose levels (153.9 ± 31.8 mg/dL and 153.6 ± 26.2 mg/dL before and after the switch, respectively; p = 0.959) (https://www.socscistatistics.com/tests/signedranks/default2.aspx).

Similarly, the standard deviation of daily blood glucose concentrations decreased from 2.97 ± 1.21 to 2.60 ± 0.63 mg/dL (p = 0.55) after switching to fast-acting insulin aspart therapy; however, the change was not significant.

Average daily risk range (ADRR) also exhibited a non-significant decreasing trend after switching to fast-acting insulin aspart therapy (from 24.45 ± 13.71 mg/dl to 20.89 ± 14.72 mg/dl (p = 0.66)). Other parameters of glycemic fluctuations did not show any significant changes.

Statistical analysis

Continuous variables were expressed using descriptive statistics (n, mean ± SD; hence, the data was examined using t-test), whereas discrete/categorical variables were summarized using frequency tables (n, %). We performed a paired t-test using the online calculator available at https://www.graphpad.com/quickcalcs/ttest1/ for an assessment of the indices of glycemic variability before and after therapy switch during (follow-up period) and for conducting a Wilcoxon signed-rank test (Table 2).

**Table 2:** Glycemic variability parameters MAGE: mean amplitude of glucose excursion, CONGA: continuous overall net glycemic action, SD: standard deviation, NS: not significant; *p < 0.05

	Paired t-test			Wilcoxon signed-rank test for GV parameters		
Parameters	Patients on RHI	Patients on fast-acting insulin aspart	P-value	W-values	Mean difference	P-value
MAGE	7.01 ± 2.59	4.19 ± 1.06	0.012*	0	1.28	Significant
SD	2.97 ± 1.21	2.60 ± 0.63	0.55	14	0.68	NS
ADDR	24.45 ± 13.71	20.89 ± 14.72	0.66	12	1.91	NS
J INDEX	49.11 ± 24.28	55.86 ± 45.40	0.75	16	1.2	NS
CONGA	8.15 ± 1.53	9.38 ± 4.15	0.4916	13	1.1	NS

A significant reduction in 24-h (nine versus five events) and night-time hypoglycemic events (five versus one event) was observed in patients after the switch to mealtime fast-acting insulin aspart therapy during the four-day follow-up period. A total of 25% of patients experienced hypoglycemic events after shifting to fast-acting insulin aspart therapy (Table 3).

**Table 3:** Frequency of hypoglycemic events RHI: regular human insulin

Hypoglycemic events	Patients on RHI therapy (before switch)	Patients on fast-acting insulin aspart therapy (after switch)	% Reduction
24-h	9	4	55%
Daytime	4	3	25%
Nighttime	5	1	80%

The patients subjected to fast-acting insulin aspart therapy exhibited a reduction in insulin dose (20.75 IU/day) as compared to patients subjected to RHI therapy (24.25 IU/day); however, the reduction in insulin dose was not significant.

## Discussion

The improved glucose variability observed in the fast-acting insulin aspart group might be attributed to its rapid onset of action and better coverage of glycemic excursion, as indicated by a significant reduction in the MAGE parameter. An improvement in glycemic fluctuation was observed especially in patients with impaired renal function in this study, which is commonly associated with the worsening of glycemic fluctuation.

Fast-acting insulin aspart is the first of the next generation faster-acting mealtime insulin analogs available for daily clinical practice in India since March 2017 [4]. This approach is characterized by the more rapid appearance of insulin in the blood after subcutaneous administration and better coverage of the mealtime excursion in glucose. Clinical studies have shown that insulin aspart exhibits twice as rapid onset of action and reaches a higher peak as compared to soluble human insulin [5].

In the present study, we found that switching the therapy from RHI to fast-acting insulin aspart significantly reduced intra-day fluctuations in glucose levels as evaluated by MAGE and frequencies of 24-hr and nighttime hypoglycemia events. Despite similar evening doses of glargine insulin, these results confirmed that fast-acting insulin aspart contributes less to insulin supply during the night as compared to human regular insulin.

Although insulin is considered the best agent to improve glycemic regulation in patients with renal failure, there is limited data about the differences in insulin profiles and their impact on minimizing GV. Therefore, a careful evaluation of its efficacy is required in patients with complications. In this study, we used CGM to compare the blood glucose stabilizing effects between both therapy groups in the presence of advanced renal insufficiency.

Heller et al. compared the effects of the rapid-acting insulin analog, insulin aspart, and soluble human insulin on hypoglycemia and glycemic regulation in patients with type 1 diabetes when injected immediately before meals as part of intensive insulin therapy. The frequency of major nighttime hypoglycemic episodes was 72% lower in patients administered with insulin aspart as compared to patients administered with human insulin (p = 0.001), which was consistent with the results of the present study. Thus, it can be concluded that the rapid-acting insulin analogs could potentially be used against major nocturnal hypoglycemic events [6]. In addition, patients administered with fast-acting aspart exhibited a lower frequency of nocturnal hypoglycemia as compared to patients administered with insulin aspart as shown via a pooled post hoc analysis (estimated treatment response 0.84; 95% CI 0.72, 0.98) [7].

With respect to the glycemic lowering efficacy, there was no major difference, as shown in a previous study, in 30 prepubertal children with type 1 diabetes using insulin glargine as their basal insulin. Diabetic children were randomly administered with either insulin aspart two minutes before meals or RHI 30 min prior to eating. At 18 weeks, the mean daily blood glucose level and glucose variability were similar in both groups (P > 0.24) [8].

There were a few limitations to this study. This article presents a report from eight patients and thus did not have any comparison group, however, patients were compared as per a pre-switch and post-switch basis. The study was conducted as a retrospective, one-arm, observational study; in addition, the sample size was small with short-duration CGM assessment. Recent consensus suggested that a minimum of 14 days of data must be collected for an accurate analysis [9]. Moreover, there were a few issues regarding the application of GV assessment. Since clinical studies about GV are scarce, more studies are needed to further elucidate the normal range and the treatment target of GV indices.

## Conclusions

The present case series demonstrated an improvement in glycemic variability following fast-acting insulin aspart therapy compared to RHI therapy as indicated by a significant decrease in MAGE, suggesting that modern ultra-rapid acting insulin bolus replacement might prove to be a useful therapeutic strategy for type 2 diabetes patients with advanced CKD. The results indicate a potential long-term impact on cardiovascular risks. The present case series sheds light on the clinical effectiveness of novel mealtime ultra-rapid-acting insulin administration approach in patients with an advanced stage of renal dysfunction and may trigger a reconsideration of the use of patient-centered insulin therapy in such patients that are difficult to treat or to be explored in future trials.
